# Acutely increasing δGABA_A_ receptor activity impairs memory and inhibits synaptic plasticity in the hippocampus

**DOI:** 10.3389/fncir.2013.00146

**Published:** 2013-09-17

**Authors:** Paul D. Whissell, Dave Eng, Irene Lecker, Loren J. Martin, Dian-Shi Wang, Beverley A. Orser

**Affiliations:** ^1^Institute of Medical Science, University of TorontoToronto, ON, Canada; ^2^Department of Pharmacology, University of TorontoToronto, ON, Canada; ^3^Department of Physiology, University of TorontoToronto, ON, Canada; ^4^Department of Anesthesia, University of TorontoToronto, ON, Canada; ^5^Department of Anesthesia, Sunnybrook Health Sciences CentreToronto, ON, Canada

**Keywords:** extrasynaptic GABA_A_ receptors, δ subunit, tonic inhibition, THIP, memory, long-term potentiation, dentate gyrus, CA1

## Abstract

Extrasynaptic γ-aminobutyric acid type A (GABA_A_) receptors that contain the δ subunit (δGABA_A_ receptors) are expressed in several brain regions including the dentate gyrus (DG) and CA1 subfields of the hippocampus. Drugs that increase δGABA_A_ receptor activity have been proposed as treatments for a variety of disorders including insomnia, epilepsy and chronic pain. Also, long-term pretreatment with the δGABA_A_ receptor–preferring agonist 4,5,6,7-tetrahydroisoxazolo[5,4-c]pyridin-3-ol (THIP) enhances discrimination memory and increases neurogenesis in the DG. Despite the potential therapeutic benefits of such treatments, the effects of acutely increasing δGABA_A_ receptor activity on memory behaviors remain unknown. Here, we studied the effects of THIP (4 mg/kg, i.p.) on memory performance in wild-type (WT) and δGABA_A_ receptor null mutant (Gabrd^−/−^) mice. Additionally, the effects of THIP on long-term potentiation (LTP), a molecular correlate of memory, were studied within the DG and CA1 subfields of the hippocampus using electrophysiological recordings of field potentials in hippocampal slices. The results showed that THIP impaired performance in the Morris water maze, contextual fear conditioning and object recognition tasks in WT mice but not Gabrd^−/−^ mice. Furthermore, THIP inhibited LTP in hippocampal slices from WT but not Gabrd^−/−^ mice, an effect that was blocked by GABA_A_ receptor antagonist bicuculline. Thus, acutely increasing δGABA_A_ receptor activity impairs memory behaviors and inhibits synaptic plasticity. These results have important implications for the development of therapies aimed at increasing δGABA_A_ receptor activity.

## Introduction

γ-Aminobutyric acid type A (GABA_A_) receptors are the primary mediators of inhibitory neurotransmission in the mammalian central nervous system. These transmitter-gated ion channels are constituted from a wide array of subunits (α1–6, β1–3, γ1–3, δ, π, θ, ε) and mediate two distinct forms of inhibition: phasic and tonic (Farrant and Nusser, 2005). Phasic inhibition is generated by postsynaptic GABA_A_ receptors, whereas tonic inhibition is mediated primarily by extrasynaptic GABA_A_ receptors that contain either the δ subunit (δGABA_A_ receptors) or α5 subunit (α5GABA_A_ receptors) (Farrant and Nusser, 2005). Recently, δGABA_A_ receptors have attracted considerable attention as therapeutic targets because these receptors significantly reduce neuronal excitability *in vitro* (Stell et al., 2003; Maguire et al., 2009) and also regulate neurogenesis (Whissell et al., 2013), memory (Wiltgen et al., 2005; Shen et al., 2010; Whissell et al., 2013), nociception (Bonin et al., 2011), maternal behaviors (Maguire and Mody, 2008) and responses to stress (Shen et al., 2007; Sarkar et al., 2011).

Drugs that directly activate δGABA_A_ receptors, and those that act as positive allosteric modulators, are currently under investigation as potential treatments for a wide variety of disorders, including insomnia (Wafford and Ebert, 2006), pain (Bonin et al., 2011), cognitive dysfunction (Wang et al., 2007) and depression (Maguire and Mody, 2008; Christensen et al., 2012). The most widely studied of these compounds is 4,5,6,7-tetrahydroisoxazolo[5,4-c]pyridin-3-ol (THIP), a δGABA_A_ receptor–preferring agonist (Brown et al., 2002; Meera et al., 2011). THIP is considered a “super”-agonist of δGABA_A_ receptors as the drug generates a greater peak response than GABA (Brown et al., 2002). The hypnotic properties of THIP were shown in studies of humans and laboratory animals (Faulhaber et al., 1997; Wafford and Ebert, 2006), and antinociceptive properties were observed in rodent models of acute and persistent pain (Bonin et al., 2011). Unlike other less selective positive modulators of GABA_A_ receptors such as benzodiazepines and barbiturates, THIP may have a low risk of tolerance and addiction (Ebert et al., 2008; Tan et al., 2011) and thus is a promising candidate for long-term use.

THIP may also have memory-enhancing effects. We recently showed that pre-treatment with THIP for 7 days improved discrimination memory, when studied 14 days after drug treatment in a mouse model (Whissell et al., 2013). The memory-enhancing properties of THIP were associated with increased postnatal neurogenesis in the dentate gyrus (DG), a process whereby new cells are generated in the adult brain. Such adult-born neurons are thought to contribute to multiple forms of memory performance, including spatial memory, recognition memory and fear memory (Marin-Burgin and Schinder, 2012).

While long-term pre-treatment with THIP improves memory, several lines of evidence predict that an acute increase in δGABA_A_ receptor activity will impair memory. First, enhanced δGABA_A_ receptor activity constrains neuronal firing (Bonin et al., 2011), reduces network excitability (Maguire et al., 2009) and attenuates synaptic plasticity in the CA1 region of the hippocampus (Shen et al., 2010). Second, one of the primary molecular targets of THIP, the α4βδ GABA_A_ receptor (Brown et al., 2002), constrains fear-associated memory (Wiltgen et al., 2005) as evidenced by studies of transgenic mice that lack either the δ subunit gene (Wiltgen et al., 2005) or the α4 subunit gene (Moore et al., 2010; Cushman et al., 2011). Interestingly, human studies have shown that THIP does not alter memory performance measured 12–24 h after drug treatment (Mathias et al., 2005; Boyle et al., 2009; Leufkens et al., 2009). However, these studies examined memory at a time point when THIP was likely to have been eliminated (Cremers and Ebert, 2007).

Here, we tested the hypothesis that acutely increasing δGABA_A_ receptor activity impairs memory. Memory was studied in wild-type (WT) and δ subunit null mutant (Gabrd^−/−^) mice 30 min after treatment with THIP, a time point when THIP levels in the brain peak (Cremers and Ebert, 2007). Additionally, to identify the molecular basis of memory impairment, long-term potentiation (LTP), a putative molecular substrate of memory, was studied in the DG and CA1 subfields of the hippocampus. A decrease or increase in GABA_A_ receptor activity enhances or depresses LTP, respectively (Wigstrom and Gustafsson, 1985; Snyder et al., 2001; Arima-Yoshida et al., 2011). Further, it has been demonstrated that selectively increasing tonic inhibition depresses LTP, even when synaptic inhibition remains unchanged (Arima-Yoshida et al., 2011). Given that δGABA_A_ receptors are densely expressed in the DG, and also expressed in the CA1 subfield (Glykys et al., 2008), it was predicted that THIP would depress LTP. Consistent with our hypotheses, the results show that acutely increasing δGABA_A_ receptor activity impairs memory, and inhibits LTP in hippocampal slices from WT but not Gabrd^−/−^ mice.

## Materials and methods

### Animals

All experiments were approved by the local Animal Care Committee. WT and Gabrd^−/−^ mice were generously provided by Dr. Gregg Homanics (University of Pittsburgh) and were generated as previously described (Mihalek et al., 1999). These mice were bred in the animal facility at University of Toronto. Only male mice 3–6 months of age were used for behavioral experiments, as the estrous cycle influences δGABA_A_ receptor expression and activity (Maguire et al., 2005). Researchers were blinded to the genotype and drug conditions.

### Drugs

THIP was obtained from Tocris Bioscience (Bristol, UK). For behavioral experiments, THIP (4 mg/kg) was administered by intraperitoneal (i.p.) injection. This dose was selected because it has no sedative effects, although it may have a mild antinociceptive effect (Bonin et al., 2011). In electrophysiological experiments, hippocampal slices were treated with 1 μM THIP, as this concentration is expected to preferentially activate δGABA_A_ receptors (Brown et al., 2002) and is within the range of the dose used to treat humans (Schultz et al., 1981; Madsen et al., 1983). The non-selective competitive GABA_A_ receptor antagonists bicuculline methiodide (BIC) and SR-95531 were employed for some experiments (Bai et al., 2001; Nusser and Mody, 2002) as no selective δGABA_A_ receptor antagonists are available. Both compounds were obtained from Tocris Bioscience (Bristol, UK). SR-95531 preferentially blocks synaptic rather than extrasynaptic GABA_A_ receptors at low concentrations (Nusser and Mody, 2002).

### Behavioral assays

#### Morris water maze

This assay was used to assess hippocampus-dependent spatial memory. The water maze was a circular pool (ø = 1.2 m) that was surrounded by visual cues, filled with opaque white non-toxic paint and kept at 25 ± 2°C. The escape platform was a 10 × 10 cm square of Plexiglas that was positioned 0.5 cm below the pool surface so that it was not visible during the experiment. On the training day, the platform was placed in a random quadrant of the pool, and mice were given 4 trials to learn its location for memory acquisition. The total time (s) to locate and remain on the platform (escape latency) was recorded during each trial. If a mouse did not locate the platform within 60 s, it was gently guided to the platform, and the maximum value of 60 s was assigned. The next day, 24 h after acquisition, long-term recall of the platform location was tested using a 60-s probe trial. During this trial, the platform was removed and the percentage of time the mouse spent in the quadrant that formerly contained the platform (the “correct” quadrant) was calculated. Mouse position was recorded and analyzed using SMART video tracking software (San Diego Instruments, San Diego, CA, USA).

#### Fear conditioning

This assay was used to examine hippocampus-dependent contextual fear memory (Phillips and LeDoux, 1992) and amygdala-dependent auditory-cued fear memory that does not require the hippocampus (Phillips and LeDoux, 1992). An exposure chamber (20 × 20 × 30 cm) with a shock grid floor consisting of stainless steel bars (2 cm apart, ø = 2 mm) was used for this task (Med Associates Inc., St. Albans, VT, USA) (Wang et al., 2012). During acquisition, each mouse was allowed to explore the chamber for 180 s. A 4 kHz tone, created by a frequency generator, amplified to 100 dB and lasting 20 s, was then presented. The last 2 s of the auditory tone was paired with an electric footshock (0.7 mA). This tone–shock pairing was presented three times (designated S1, S2, S3), separated by 60-s intervals. The next day (i.e., day 2), 24 h after acquisition, contextual fear memory was assessed by returning the mouse to the context for 8 min and measuring the percentage of time that it spent freezing. On day 3, the conditioning chamber was modified to measure the freezing response to the auditory tone (auditory-cued fear memory). This modified context had a significantly different shape, scent and visual appearance than the original chamber. Mice were monitored for 180 s for freezing to the modified context, to rule out contextual influences. After this monitoring period, the auditory tone was presented for 5 min, and the percentage of time that each mouse spent freezing was determined using FreezeView software (Version 2.26, Actimetrics Inc., Wilmette, IL, USA).

#### Novel object recognition

This assay was used to study short-term working memory. Twenty-four hours before testing, each mouse was habituated for 15 min in a chamber (20 × 20 × 20 cm) marked with visual cues (Saab et al., 2009). During testing, the mouse was exposed to a set of three identical objects in the chamber for 2 min (Figure 3A). The mouse was then removed from the chamber for 2 min while the entire setup was cleaned with 70% ethanol and one of the objects was replaced with a novel object (NO). The mouse was then returned to the chamber and the interaction time with the two familiar objects (O1 + O2) and the NO was recorded. Total interaction time was the sum of these interaction times (O1 + O2 + NO). NO preference (%) was defined as NO/(O1 + O2 + NO) × 100. An interaction was defined as active investigation of the object while the mouse was within 1 cm of the object and oriented toward it. Mice with a total interaction time of less than 3 s were excluded from analysis.

### Electrophysiology

Male mice were anesthetized deeply with isoflurane and then decapitated, and their brains were removed. Coronal hippocampal slices (350–400 μm thick) were cut with a vibratome (VT1000E; Leica, Deerfield, IL, USA), then immersed in ice-cold artificial cerebrospinal fluid (ACSF) that contained (in mM) 124 NaCl, 3 KCl, 1.3 MgCl2, 2.6 CaCl2, 1.25 NaH2PO4, 26 NaHCO3, and 10 d-glucose. The ACSF was saturated with 95% O2 and 5% CO2, and osmolarity was adjusted to 300–310 mOsm. The slices were allowed to recover for at least 1 h at room temperature (23–25°C) before being transferred to the recording chamber, where they were perfused with ACSF at 3–4 ml/min. All recordings were performed at room temperature using a MultiClamp 700A amplifier (Molecular Devices, Sunnyvale, CA, USA) controlled with pClamp 9.0 software via a Digidata 1322A interface (Molecular Devices, Sunnyvale, CA, USA).

#### Extracellular field potential recordings

Hippocampal slices were obtained from 3- to 6-month-old mice. In experiments examining LTP in the DG, extracellular field postsynaptic potentials (fPSPs) were recorded from the stratum moleculare of the DG using an ACSF-filled borosilicate pipette (World Precision Instruments, Sarasota, FL, USA). The medial perforant pathway was stimulated with a bipolar tungsten electrode (Rhodes Medical Instruments, Summerland, CA, USA).

To study presynaptic plasticity and confirm correct placement of the stimulating electrode in the medial perforant pathway, a pair of stimuli was applied at various time intervals (50, 100, 150, 200, or 300 ms) to generate a pair of responses. The paired pulse ratio was defined as (the slope of response 2)/(the slope of response 1). The presence of paired pulse depression (ratio < 1), one of the criteria used to assess medial perforant pathway inputs (Christie and Abraham, 1994), was deemed to indicate successful stimulation of the medial perforant pathway. To record LTP, baseline fPSPs were measured for at least 10 min at 0.05 Hz using a stimulation intensity that produced a half-maximal response. LTP was induced with a stimulation protocol that consisted of 4 stimulus trains delivered every 20 s, with each train occurring at 100 Hz and lasting 500 ms. fPSPs were monitored for 60 min after the stimulation, and the average of the last 5 min of recording was compared with the average of the baseline fPSPs. All drugs were allowed to perfuse the slices for 15 min before recording.

In experiments examining LTP in CA1, the same procedure was followed except that the recording electrode was placed in the stratum radiatum of the CA1 subfield and the Schaffer collateral pathway was stimulated. The protocol for LTP induction consisted of 10 stimulus trains of 4 pulses at 100 Hz with an inter-train interval of 500 ms (Martin et al., 2009).

#### Whole-cell voltage-clamp recordings

Hippocampal slices were obtained from 14- to 21-day-old male mice. Mice of this age range were utilized as their brains exhibit are more resistant to the dissection process (Moyer and Brown, 1998) and offer a larger population of healthy cells for easier patching. These cells show significant δGABA_A_ receptor expression and δGABA_A_ receptor-mediated currents (Laurie et al., 1992; Shen et al., 2011). All recordings were obtained from cells located in the granule cell layer of the DG that were visually identified with a Olympus BX51WI microscope (Center Valley, PA, USA). Recording pipettes (3–5 MΩ) were filled with the intracellular solution containing (in mM) 140 CsCl, 11 ethylene glycol tetra-acetic acid, 10 4-(2-hydroxyethyl)-1-piperazineethanesulfonic acid, 2 K2-ATP, 1 CaCl2, 2 MgCl2 and 2 tetraethylammonium with osmolarity adjusted to 290–295 mOsm and pH adjusted to 7.3. To block glutaminergic neurotransmission and voltage-dependent sodium channels, 6-Cyano-7-nitroquinoxaline-2,3-dione (10 μM), (2R)-amino-5-phosphonovaleric acid (40 μM), and tetrodotoxin (0.5 μM) were added to the ACSF. All recordings were performed at a holding potential of –70 mV, sampled at 10 kHz and filtered at 2 kHz by a low-pass Bessel filter. Cells were included in analysis only if they had an access resistance of ≤ 20 MΩ and this resistance did not vary by more than 20% during the recording period.

### Statistical analyses

Statistical analyses were conducted using Graphpad Prism 5.0 and SPSS17 for Windows. The acquisition data for the Morris water maze and fear conditioning assay were analyzed using repeated-measures analysis of variance (ANOVA). In other cases, either a Student's *t*-test or a standard One-Way or Two-Way ANOVA followed by Bonferroni *post-hoc* test was used. All values are expressed as mean ± SEM, and *p* < 0.05 was considered statistically significant. Performance scores more than 2 standard deviations from the mean were excluded from the analysis.

## Results

### THIP impairs spatial memory in the morris water maze

**Figure 1:**
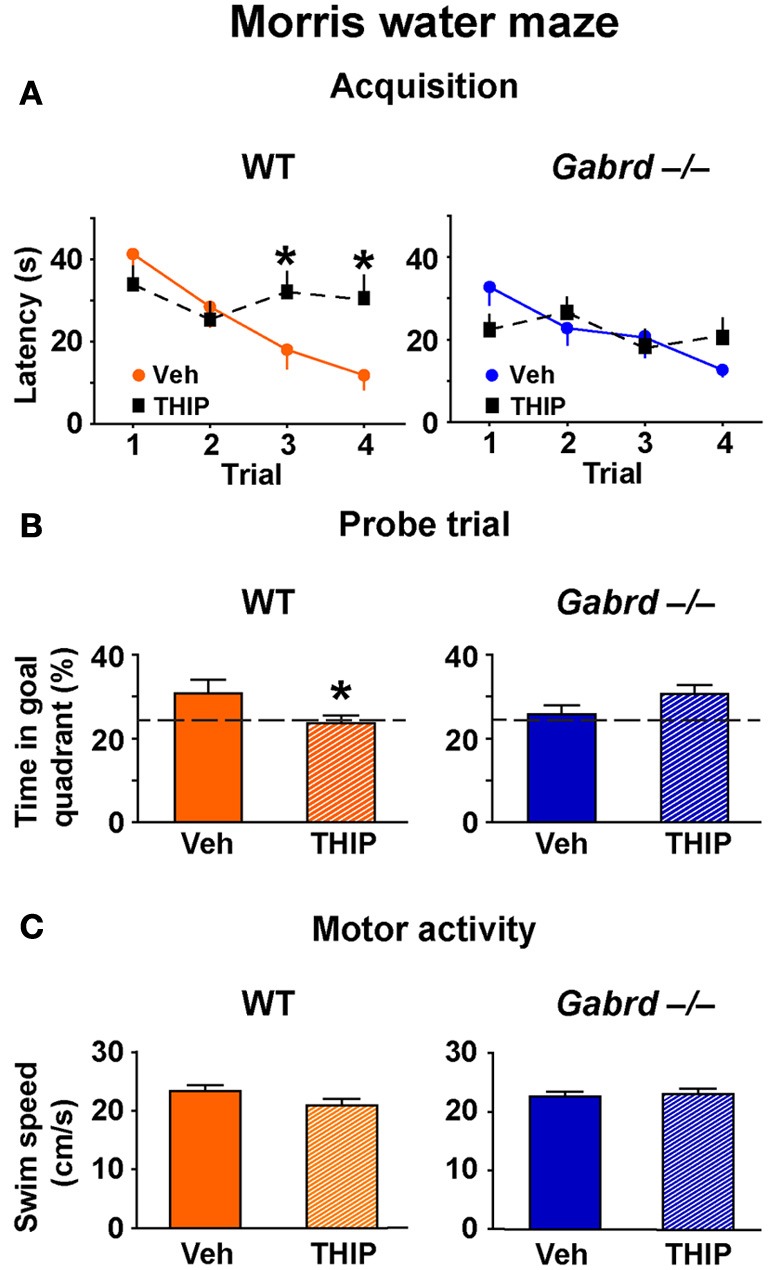
**THIP impairs spatial memory in the Morris water maze. (A)** THIP increased the escape latencies on the third and fourth trials in WT but not Gabrd^−/−^ mice. **(B)** THIP decreased the preference for the goal quadrant formerly containing the escape platform in WT but not Gabrd^−/−^ mice. **(C)** THIP did not affect swim speed in WT or Gabrd^−/−^ mice. *n* = 16–19, ^*^p < 0.05.

The effect of THIP on spatial memory was first examined in the Morris water maze, as performance of this task is hippocampus-dependent and is regulated by GABA_A_ receptor activity (D'Hooge and De Deyn, 2001; Collinson et al., 2002; Myhrer, 2003; Cheng et al., 2006). WT and Gabrd^−/−^ mice were treated with THIP (4 mg/kg, i.p.) or vehicle 30 min before being trained over 4 trials to locate a hidden platform for memory acquisition. All mice learned to locate the platform, as evidenced by reduced escape latencies over sequential trials, and there were no baseline differences in acquisition between vehicle-treated WT and Gabrd^−/−^ mice (Figure 1A; genotype × trial, *p* > 0.2, *n* = 16–19). Notably, THIP-treated WT mice showed impaired acquisition relative to vehicle-treated WT mice, as evidenced by slower escape latencies on the third and fourth trials (Figure 1A, left; genotype × drug × trial, *p* < 0.05). In contrast, THIP had no effect in Gabrd^−/−^ mice (Figure 1A, right).

Next, to investigate whether THIP impairs long-term memory, recall of the platform location was tested in a probe trial that was performed 24 h after the acquisition trials. THIP-treated WT mice spent less time in the target quadrant that formerly contained the platform compared with vehicle-treated WT mice (Figure 1B, left; genotype × drug, *p* < 0.05, *n* = 14–17). In contrast, THIP- and vehicle-treated Gabrd^−/−^ mice performed similarly (Figure 1B, right). THIP had no effect on motor activity in any of the groups, as swim speed was unchanged (Figure 1C; drug and drug × genotype, all *p*-values > 0.05). Collectively, these results indicate that THIP impaired spatial memory in WT but not Gabrd^−/−^ mice.

### THIP impairs contextual but not auditory-cued fear memory

**Figure 2:**
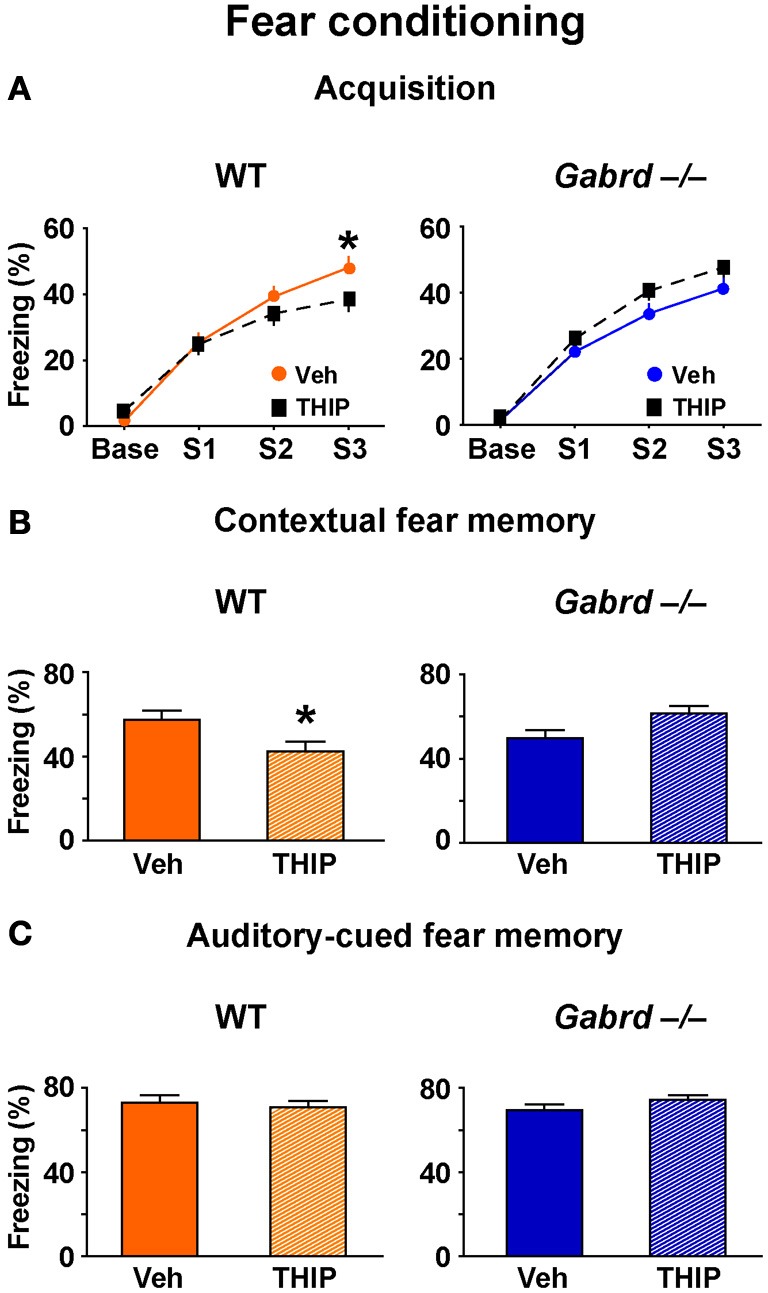
**THIP impairs contextual but not auditory-cued fear memory. (A)** THIP decreased the freezing score following the third tone-shock pairing in WT but not Gabrd^−/−^ mice. **(B)** THIP reduced the freezing score for contextual fear memory in WT but not Gabrd^−/−^ mice. **(C)** THIP did not affect the freezing score for auditory-cued fear memory in response to tone in both WT and Gabrd^−/−^ mice. *n* = 25–30, ^*^p < 0.05.

To determine whether increased activity of δGABA_A_ receptors regulates additional forms of hippocampus-dependent memory, the effect of THIP on aversive contextual fear conditioning (Phillips and LeDoux, 1992) was studied. Thirty minutes after injection, mice were trained to associate an electric footshock (an unconditioned stimulus) with a context and auditory cue (conditioned stimuli). A 1-day acquisition protocol that utilized three mild footshocks (designated S1, S2, and S3) was employed (Mihalek et al., 1999). During acquisition, all groups showed progressively increasing levels of freezing after each shock (Figure 2A), indicating they successfully acquired the task. There was no difference in acquisition between vehicle-treated WT and Gabrd^−/−^ mice (genotype × shock, *p* > 0.5, *n* = 25–30), which is consistent with previous results obtained with this protocol (Mihalek et al., 1999). However, THIP-treated WT mice exhibited reduced freezing after the third shock compared with vehicle-treated controls (Figure 2A, left; genotype × drug × shock, *p* < 0.01, *n* = 25–30). In contrast, THIP had no effect on Gabrd^−/−^ mice (Figure 2A, right). These results indicate that THIP impaired the acquisition of fear memory.

To measure contextual fear memory, mice were returned to the same training context 24 h after fear acquisition. THIP-treated WT mice showed reduced freezing scores relative to vehicle-treated controls, indicating reduced contextual fear memory (Figure 2A, left; genotype × drug, *p* < 0.01, *n* = 25–30). Gabrd^−/−^ mice treated with THIP exhibited no memory deficits (Figure 2B, right). Next, the effects of THIP on auditory-cued fear memory, an amygdala-dependent task that does not normally require the hippocampus (Phillips and LeDoux, 1992), was examined. Interestingly, THIP did not impair auditory-cued fear memory in WT or Gabrd^−/−^ mice (Figure 2C; *p* > 0.2 for main effects and interaction, *n* = 25–30). Collectively, these results show that THIP impairs contextual but not auditory-cued fear memory.

### THIP impairs novel object recognition

**Figure 3:**
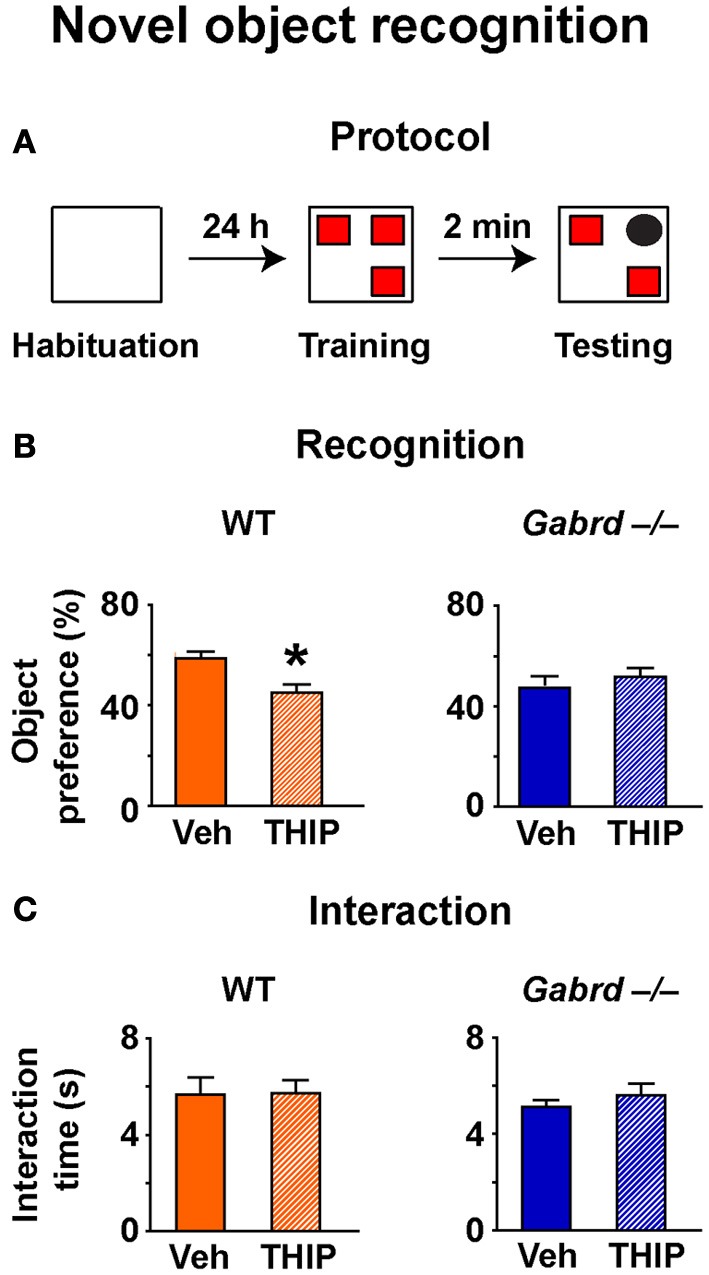
**THIP impairs novel object recognition. (A)** Schematic diagram showing the protocol. **(B)** THIP decreased the preference for the novel object in WT but not Gabrd^−/−^ mice. **(C)** THIP had no effect on total interaction time in both WT and Gabrd^−/−^ mice. *n* = 12–21, ^*^p < 0.05.

To determine whether increased δGABA_A_ receptor activity impairs short-term working memory, the NO recognition task was used. In this assay, mice must recognize a NO within a set of familiar objects to which they have been previously exposed (Figure 3A). Because animals are driven to investigate novelty, mice that recall the familiar objects will preferentially interact with the NO (Ennaceur and Meliani, 1992). NO recognition is a non-aversive task that depends primarily upon the perirhinal cortex (Winters et al., 2010) and is regulated by GABA_A_ receptors (Zurek et al., 2012; Whissell et al., 2013).

NO preference was reduced in vehicle-treated Gabrd^−/−^ mice relative to vehicle-treated WT mice (Figure 3B; WT + vehicle = 59.4 ± 2.2%, Gabrd^−/−^ + vehicle = 47.6 + 3.2%; drug × genotype, *p* < 0.05, *n* = 12–21), a result that is consistent with our previous finding (Whissell et al., 2013). THIP-treated WT mice showed impaired NO recognition relative to vehicle-treated controls (Figure 3B, left; drug × genotype, *p* < 0.05, *n* = 12–21), whereas THIP had no effect in Gabrd^−/−^ mice (Figure 3B, right). These results could not be attributed to an effect of either the genotype or THIP treatment on exploratory drive, as the total object interaction time was similar in all groups (Figure 3C).

### THIP depresses long-term potentiation in the dentate gyrus

**Figure 4:**
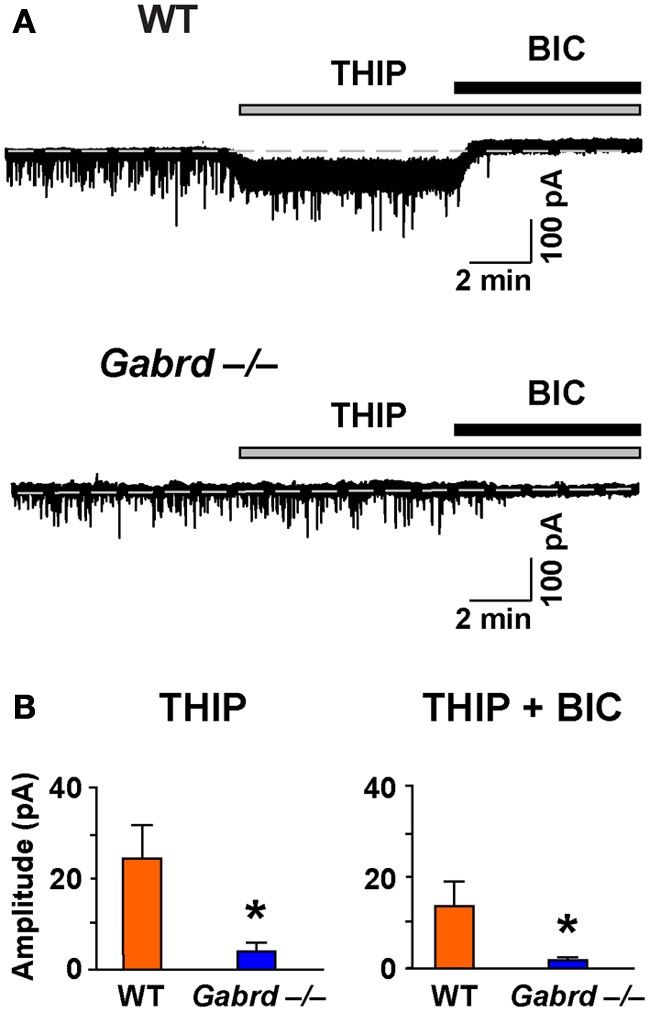
**The effects of THIP, and the tonic inhibitory current revealed by BIC, in DG granule cells from WT and Gabrd^−/−^ mice. (A)** Representative recordings show the effects of THIP, and the tonic current revealed by BIC (20 μM). **(B)** Quantified data. *n* = 6–7, ^*^p < 0.05.

We next examined the effects of THIP on LTP in the DG of the hippocampus as δGABA_A_ receptors are densely expressed in this region (Pirker et al., 2000). Before studying LTP, we confirmed that THIP (1 μM) increased a tonic δGABA_A_ receptor–mediated conductance using whole-cell voltage-clamp recordings. Perfusion of THIP activated a significant inward current in granule cells from WT but not Gabrd^−/−^ mice (Figure 4; *p* < 0.01, *n* = 6–7). The competitive GABA_A_ receptor antagonist BIC (20 μM) completely blocked the effects of THIP. BIC also reduced the baseline holding current and revealed a tonic conductance generated by GABA_A_ receptors. The tonic current was greater in WT neurons than Gabrd^−/−^ neurons (Figure 4; *p* < 0.05, *n* = 6–7).

**Figure 5:**
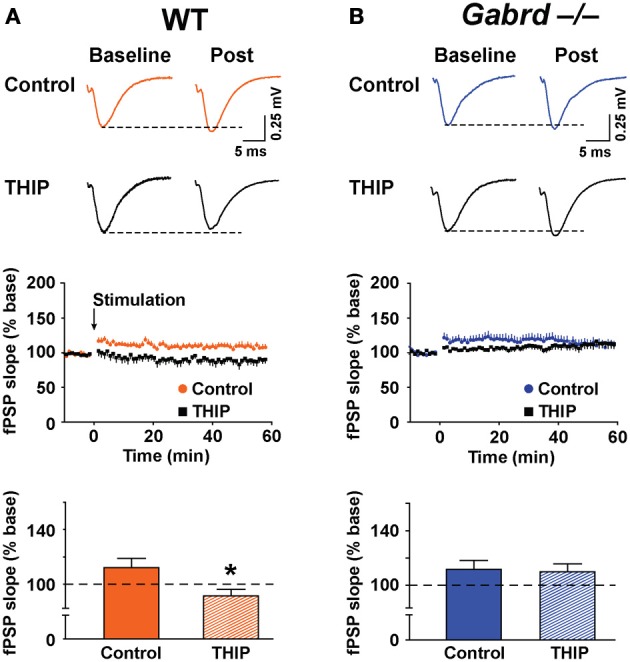
**THIP inhibits long-term potentiation in the dentate gyrus. (A,B)** THIP depressed LTP in the DG in slices from WT but not Gabrd^−/−^ mice. Upper panels: Representative traces before and after tetanic stimulation. Middle panels: Normalized slope of fPSPs following tetanic stimulation. Bottom panels: Summarized data showing the last 5 min of recording. Note that THIP depressed LTP in DG only in WT mice. *n* = 8–13, ^*^p < 0.05.

The effect of THIP on LTP obtained in the stratum moleculare of the DG was examined in slices from WT and Gabrd^−/−^ mice following tetanic stimulation of the medial perforant pathway. The slope of fPSPs after stimulation increased to 112.2 ± 6.7% (*n* = 10) and 110.0 ± 6.5% (*n* = 10) of baseline in WT and Gabrd^−/−^ slices, respectively (Figure 5). There was no difference in the amplitude of LTP between genotypes (*p* > 0.4). Interestingly, THIP treatment completely blocked LTP in the DG in slices from WT mice (Figure 5A; WT + THIP = 91.5 ± 4.8%; genotype × drug, *p* < 0.05, *n* = 8–10) but not from Gabrd^−/−^ mice (Figure 5B; Gabrd^−/−^ + THIP = 109.2 ± 5.7%; genotype × drug, *p* > 0.05, *n* = 10–13). These results suggest that THIP depresses LTP in the DG by acting upon δGABA_A_ receptors.

### Extrasynaptic GABA_A_ receptors mediate the inhibitory effect of THIP on LTP

**Figure 6:**
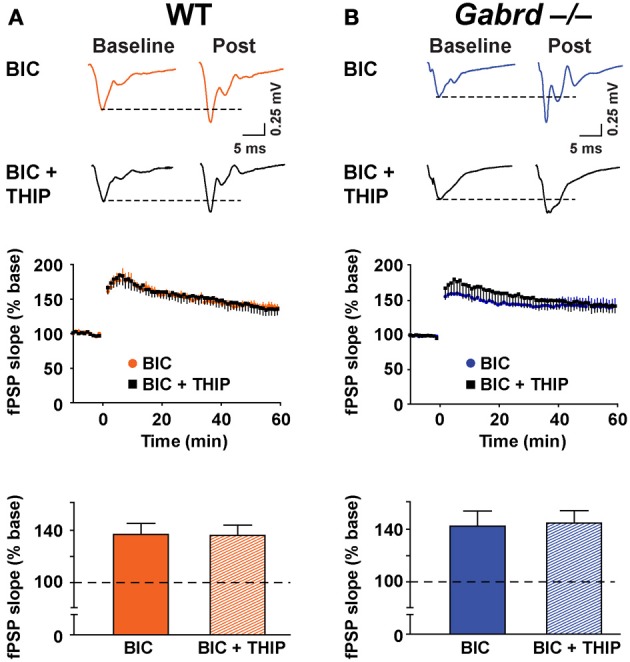
**BIC occludes the inhibitory effects of THIP on long-term potentiation in the dentate gyrus. (A,B)** THIP does not impair LTP in the DG in BIC-treated slices. BIC (100 μM) was perfused throughout the recordings. Upper panels: Representative traces before and after tetanic stimulation. Middle panels: Normalized slope of fPSPs following tetanic stimulation. Bottom panels: Summarized data showing the last 5 min of recording. Note that THIP did not depress LTP in the DG in both WT and Gabrd^−/−^ mice. *n* = 9–10.

To verify that GABA_A_ receptors were involved in THIP effects on plasticity, LTP was studied in the presence of BIC (100 μM). Application of BIC alone increased LTP to 136.7 ± 8.4% and 142.3 ± 11.7% for WT and Gabrd^−/−^ mice, respectively (Figure 6, *n* = 9–10). This marked increase in plasticity is consistent with results reported by others (Snyder et al., 2001). Co-application of THIP (1 μM) and BIC did not reduce LTP (Figure 6; WT + BIC + THIP = 135.9 ± 8.1%, Gabrd^−/−^ + BIC + THIP = 144.6 ± 9.5%; drug and drug × genotype, *p* > 0.4, *n* = 9–10) suggesting THIP actions are mediated by GABA_A_ receptors.

**Figure 7:**
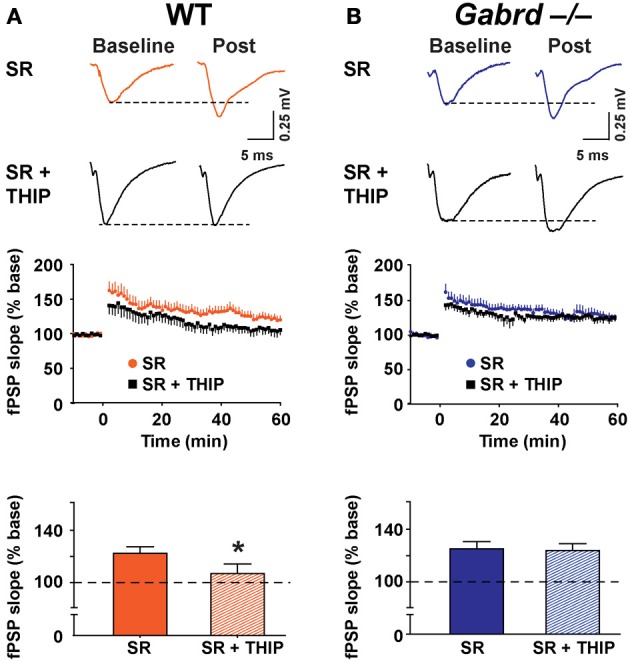
**SR-95531 does not prevent THIP-mediated depression of long-term potentiation in the dentate gyrus. (A,B)** THIP impairs LTP in the DG of SR-95531-treated slices from WT but not Gabrd^−/−^ mice. SR-95531 (1 μM) was perfused throughout the recordings. Upper panels: Representative traces before and after tetanic stimulation. Middle panels: Normalized slope of fPSPs following tetanic stimulation. Bottom panels: Summarized data showing the last 5 min of recording. *n* = 10–12. ^*^p < 0.05.

Next, LTP was studied in the presence of SR-95531 (1 μM), a compound that preferentially blocks synaptic GABA_A_ receptors at low concentrations (Nusser and Mody, 2002). Application of SR-95531 alone did not significantly elevate LTP (WT = 112.2 ± 6.7% vs. WT + SR-95531 = 123.3 ± 5.2%, Gabrd^−/−^ = 110.0 ± 6.5% vs. Gabrd^−/−^ + SR-95531 = 125.7 ± 5.4%; *p* > 0.05, *n* = 10–12) (Figures 5, 7). THIP reduced LTP in SR-95531-treated slices from WT mice (Figure 7; WT + SR-95531 = 123.3 ± 5.2% and WT + SR-95531 + THIP = 107.8 ± 7.2%, drug effect, *p* < 0.05, *n* = 10–12), but not in slices from Gabrd^−/−^ mice (Figure 7; Gabrd^−/−^ + SR-95531 = 125.7 ± 5.4%, Gabrd^−/−^ + SR-95531 + THIP = 124.4 ± 5.2%; drug effect, *p* > 0.05, *n* = 10–12). These results indicate that the inhibitory effects of THIP on LTP are mediated by extrasynaptic rather than synaptic GABA_A_ receptors.

### δGABA_A_ receptor activity does not alter baseline synaptic transmission or presynaptic function in the dentate gyrus

**Figure 8:**
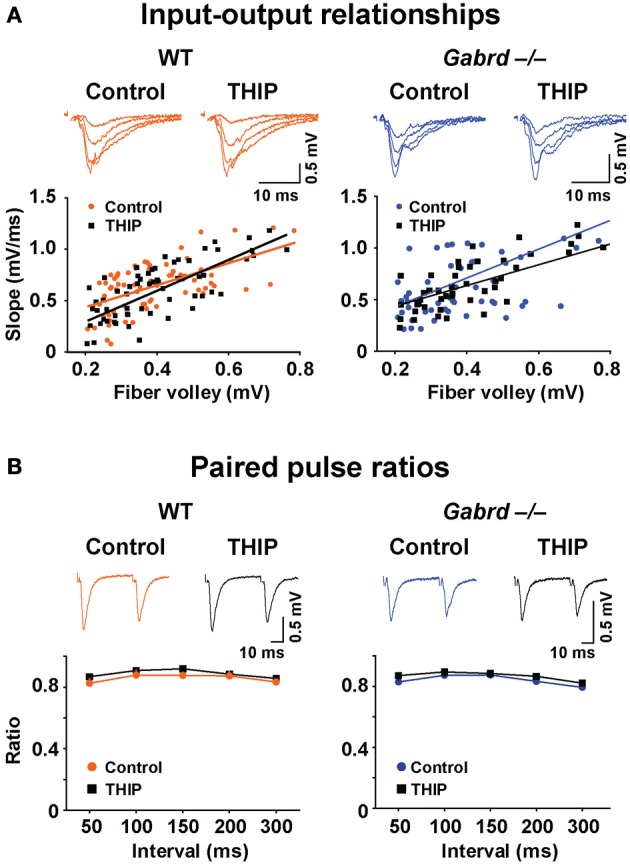
**THIP has no effects on baseline synaptic transmission or presynaptic function in slices from both WT and Gabrd^−/−^ mice. (A)** Representative traces show fPSPs with increasing stimulus intensities. The input-output relationships beneath the traces, indicators of baseline synaptic transmission, were similar between genotypes, and between control and THIP treatment groups in either genotype. *n* = 9–10. **(B)** Sample traces show paired-pulse depression. Paired pulse ratios, indicators of presynaptic function, were also similar between genotypes, and between control and THIP treatment groups in either genotype. *n* = 9–10.

We then examined whether δGABA_A_ receptors modify baseline synaptic transmission in the DG by studying the input–output relationships for field potentials recorded in WT and Gabrd^−/−^ slices. To generate an input–output plot, the stimulus intensity was increased incrementally to generate fPSPs of increasing strength. The amplitude of the presynaptic fiber volley vs. the slope of each fPSP was graphed as a scatter plot. The presynaptic fiber volley and the slope of each fPSP are indicative of presynaptic input (fiber activation) and postsynaptic output, respectively. A “best-fit line” representing the input–output relationship was then computed using linear regression (Figure 8A). There was no difference in the slope of the input–output relationship in relation to either genotype. Similarly, treating the slices with THIP did not alter the input-output relationship (all *p*-values > 0.05).

We next investigated the effects of THIP on the ratio of paired pulses in DG, which represents a presynaptic form of short-term plasticity. To generate paired pulses, two fPSPs were elicited by applying two stimuli to the medial perforant pathway at varying time intervals ranging from 50 to 300 ms. The ratio of the resulting responses was then computed (response 2/response 1). As reported previously (Christie and Abraham, 1994), paired pulse depression (ratio < 1) was observed in the DG with stimulation of the medial perforant pathway (Figure 8B). There was no difference in paired pulse ratios in relation to either genotype or THIP treatment (*p* > 0.05).

### THIP depresses long-term potentiation in the CA1 region

**Figure 9:**
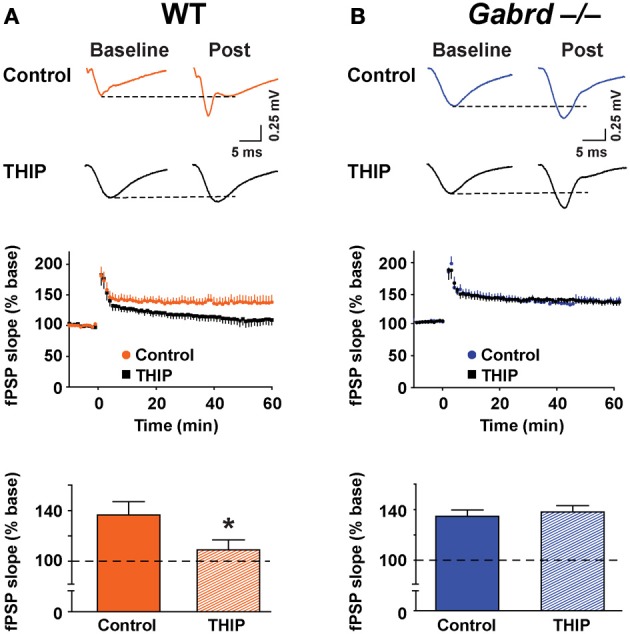
**THIP inhibits long-term potentiation in the CA1 region. (A,B)** THIP depressed LTP in CA1 in slices from WT but not Gabrd^−/−^ mice. Upper panels: Representative traces before and after tetanic stimulation. Middle panels: Normalized slope of fPSPs following tetanic stimulation. Bottom panels: Summarized data showing the last 5 min of recording. Note that THIP depressed LTP in CA1 only in WT mice. *n* = 8–9, ^*^p < 0.05.

Finally, to determine if THIP depressed plasticity in other regions of the hippocampus, LTP was studied in the CA1 subfield. δGABA_A_ receptors are expressed in this region (Pirker et al., 2000) but generate a lower magnitude current under baseline conditions when compared with the DG (Glykys et al., 2008). fPSPs were recorded in the stratum radiatum of the CA1 subfield before and after tetanic stimulation of the Schaffer collateral pathway. Stimulation increased the slope of fPSPs to 137.3 ± 10.3% (*n* = 8) and 135.4 ± 5.1% (*n* = 8) of baseline in WT and Gabrd^−/−^ mice, respectively (Figure 9). There were no differences in LTP in CA1 between genotypes (*p* > 0.1). THIP treatment attenuated LTP in slices from WT mice (Figure 9A; WT + THIP = 109.6 ± 7.9%; genotype × drug, *p* < 0.05, *n* = 8–9) but not from Gabrd^−/−^ mice (Figure 9B; Gabrd^−/−^ + THIP = 138.7 ± 5.0%; genotype × drug, *p* > 0.05, *n* = 8–9).

## Discussion

The above results show that THIP impaired multiple forms of memory in WT but not Gabrd^−/−^ mice. THIP also depressed LTP, but only in slices from WT mice. Collectively, these results show that the neurodepressive effects of THIP were mediated by δGABA_A_ receptors.

### THIP impairs memory

THIP selectively impaired hippocampus-dependent memory, as evidenced by deficits in the Morris water maze and contextual fear conditioning tasks. In contrast, auditory-cued fear conditioning, a behavioral task which primarily depends upon the amygdala rather than the hippocampus (Phillips and LeDoux, 1992), was unaffected. The vulnerability of hippocampus-dependent memory to THIP may be attributed to high expression levels of δGABA_A_ receptors in the hippocampus, relative to other brain regions involved in memory, such as the amygdala (Pirker et al., 2000).

THIP-mediated impairment of long-term memory was likely due to impaired memory acquisition. These effects of THIP are consistent with previous results as neuroactive steroids that act as positive allosteric modulators of the δGABA_A_ receptor (Belelli and Lambert, 2005) reduce memory acquisition. Specifically, allopregnanolone impairs acquisition in the shock avoidance assay (Shen et al., 2010), while tetrahydroprogesterone impairs acquisition in the Y-maze recognition task (Mayo et al., 1993). Also, muscimol and other non-selective agonists of the GABA_A_ receptor that increase δGABA_A_ receptor activity impair acquisition in multiple memory tasks (Myhrer, 2003; Makkar et al., 2010). Whether THIP also impairs the consolidation or retrieval of memory is a topic for future study.

Interestingly, NO recognition was also impaired by THIP. This behavior is primarily regulated by the perirhinal cortex (Winters et al., 2010). δGABA_A_ receptors expressed in this area (Pirker et al., 2000) and in other cortical regions (Drasbek and Jensen, 2006) may be involved in the effects of THIP. Alternatively, δGABA_A_ receptors in the hippocampus may substantially contribute to the THIP effects. The hippocampus can modify recognition memory when a novel and/or complex testing environment is used (Oliveira et al., 2010; Sannino et al., 2012) or when the interval between the training and testing phases is short (Rose et al., 2012). The current experiments utilized a complex testing environment that included multiple visual cues and three objects. Further, there was a relatively short interval between the training and testing periods (2 min). Such testing conditions may facilitate the involvement of hippocampal δGABA_A_ receptors in THIP impairment of NO recognition.

### Baseline memory is not enhanced in GABRD^−/−^ mice

Gabrd^−/−^ mice did not differ from WT mice in baseline contextual fear conditioning and Morris water maze performance. These data are consistent with previous results that showed no enhanced memory in male Gabrd^−/−^ mice (Mihalek et al., 1999; Wiltgen et al., 2005). Interestingly, the unchanged memory performance of Gabrd^−/−^ mice contrasts with the generally enhanced memory seen in other GABA_A_ receptor subunit knockout mice. Notably, transgenic mice lacking the α4 subunit (Moore et al., 2010; Cushman et al., 2011) or α5 subunit (Collinson et al., 2002; Martin et al., 2010) exhibit enhanced memory performance, particularly in the contextual fear conditioning and Morris water maze tasks. While there was no evidence of enhanced memory in Gabrd^−/−^ mice, the impairment in NO recognition is consistent with our previous report (Whissell et al., 2013).

Several potential explanations account for the lack of memory enhancement in Gabrd^−/−^ mice. The deletion of the δGABA_A_ receptor impedes neurogenesis in the DG (Whissell et al., 2013), a process that contributes to memory performance (Marin-Burgin and Schinder, 2012). Disruption of neurogenesis is associated with impaired memory performance in the Morris water maze, contextual fear conditioning and NO recognition tasks (Snyder et al., 2005; Saxe et al., 2006; Jessberger et al., 2009). Thus, reduced neurogenesis may counteract the potential enhancement of memory caused by reduction of tonic inhibition in Gabrd^−/−^ mice. Alternatively, deletion of the δ subunit may induce a compensatory change in the expression or function of other ion channels which regulate memory, such as potassium channels (Brickley et al., 2001) or α5GABA_A_ receptors (Glykys et al., 2008).

### THIP impairs long-term potentiation in the hippocampus

To identify the neurophysiological substrate of THIP-induced memory deficits, LTP was measured in the DG and CA1. LTP in the DG (~110% of baseline) was roughly one third the magnitude of LTP in the CA1 (~130% of baseline). The low LTP in the DG has been attributed to strong GABA_A_ receptor-mediated inhibition (Wigstrom and Gustafsson, 1983; Snyder et al., 2001; Arima-Yoshida et al., 2011). Consistent with this postulate, BIC enhanced LTP in the DG nearly 4-fold (from ~110 to ~140% of baseline). In contrast, only subtle and variable effects of BIC on LTP were reported in the CA1 region; BIC either enhanced LTP 2-fold (Arima-Yoshida et al., 2011) or had no significant effect (Chen et al., 2011).

Interestingly, we observed no baseline differences in LTP in either the DG or CA1 between WT and Gabrd^−/−^ slices. The lack of enhanced LTP in the DG of Gabrd^−/−^ slices might be explained by the disruption of neurogenesis (Whissell et al., 2013). Neurogenesis facilitates baseline LTP in the DG (Snyder et al., 2001) likely because adult-born neurons show greater plasticity than older or developmentally-generated neurons (Ming and Song, 2011). We also observed no increase in LTP in the CA1 in Gabrd^−/−^ mice, possibly due to the relatively low expression of δGABA_A_ receptors in this region (Pirker et al., 2000). Additionally, as discussed above, compensatory changes in the expression of other receptors that constrain plasticity, such as α5GABA_A_ receptors, might be contributing factors (Glykys et al., 2008; Martin et al., 2010).

THIP depressed LTP in the DG and CA1 in slices from WT mice but not Gabrd^−/−^ mice, which is consistent with impaired memory in THIP-treated WT mice. Others showed that LTP in the DG is impaired by increasing tonic inhibition with low concentrations GABA (Arima-Yoshida et al., 2011). THIP also significantly attenuated LTP in the CA1 region, a result that was somewhat surprising given the relatively low expression and baseline activity of δGABA_A_ receptors in this area (Pirker et al., 2000; Glykys et al., 2008). δGABA_A_ receptors in the CA1 may play a more important role in memory processes than initially thought, particularly when these receptors are highly activated by drugs. Alternatively, impairment of LTP in the CA1 might be due to activation of other, non-δGABA_A_ receptors. Low concentrations of THIP within the range employed in this study (~2 μM) also activate extrasynaptic α5GABA_A_ receptors (Ebert et al., 1997; Lindquist et al., 2003), which are present in the CA1 subfield and constrain LTP (Martin et al., 2010).

Possible mechanisms for THIP-mediated depression of LTP include membrane hyperpolarization and shunting inhibition (Andersen et al., 1980; Staley and Mody, 1992). THIP-mediated membrane hyperpolarization would be expected to impair LTP via inhibition of channels critical for LTP, such as N-methyl-D-aspartate receptors (Morris et al., 1986). Alternatively, THIP may impair LTP via shunting inhibition. The opening of δGABA_A_ receptor channels by THIP would decrease the neuronal input resistance and attenuate the membrane depolarization elicited by excitatory neurotransmitters, which would also impair LTP.

In the current study, THIP did not affect the input/output relationship, a correlate of neuronal excitability. This result contrasts with the finding that increases in δGABA_A_ receptor activity with neurosteroids shift the input-output relationship to the right (Stell et al., 2003). Methodological factors may account for this discrepancy. In this study, stimulus intensity was incrementally increased to generate output fPSPs (Martin et al., 2010). In the previous report (Stell et al., 2003), stimulus intensity was kept constant but stimulus duration (i.e., the pulse width) was incrementally increased. These two inputs (stimulus intensity vs. pulse width) may not produce similar results. In addition, different compounds with distinct mechanisms of action were employed in the two studies. THIP is a “super”-agonist of the δGABA_A_ receptor (Brown et al., 2002), whereas neurosteroids are positive allosteric modulators of the δGABA_A_ receptor (Belelli and Lambert, 2005).

### Potential therapeutic implications

Our current and previous findings (Whissell et al., 2013) show that THIP has two distinct effects on memory. A single acute treatment with THIP reduces memory performance, possibly due to increased tonic inhibitory conductance and reduced synaptic plasticity in the hippocampus. In contrast, long-term pre-treatment with THIP enhanced memory performance and neurogenesis weeks after THIP had been eliminated. THIP, administered as a single injection in the current study, was unlikely to influence neurogenesis, a process that occurs over a time period of many weeks (Zhao et al., 2008).

The acute memory-blocking properties of THIP may be desirable in several clinical contexts. For example, THIP could be used as an adjunct to facilitate the induction of general anesthesia or to prevent inadvertent recall of traumatic events during surgery (Mashour et al., 2011). Under other conditions, THIP-induced memory loss could be highly undesirable, such as during the performance of demanding memory tasks (e.g., studying) or during spatial navigation (e.g., driving) (Leufkens et al., 2009). Any long-term beneficial effects of THIP must be carefully weighed against acute effects that reduce memory performance. Future studies are required to determine an optimum dose and drug protocol that maximizes the therapeutic effects of THIP but minimizes undesired memory loss. It is also of interest to determine whether other off-target effects of THIP, such as ataxia (Bonin et al., 2011) or driving impairment (Leufkens et al., 2009), result from increased δGABA_A_ receptor activity.

Finally, the present study demonstrates significant memory-blocking properties of THIP in healthy adult male WT mice. The sensitivity to THIP may vary with age, gender, physiologic state or other factors. Notably, δGABA_A_ receptor expression is significantly increased during puberty (Shen et al., 2010), certain stages of the ovarian cycle (Maguire et al., 2005), stress (Sanna et al., 2011) and following traumatic brain injury (Kharlamov et al., 2011). Thus, the memory-blocking effects of THIP may be greatly enhanced in certain clinical populations, which is an additional consideration in the therapeutic use of this drug.

## Author contributions

Paul D. Whissell, Dave Eng, Loren J. Martin, and Beverley A. Orser designed the studies; Paul D. Whissell, Dave Eng, and Irene Lecker performed the experiments; Paul D. Whissell analyzed the data; Paul D. Whissell, Dian-Shi Wang, Irene Lecker, and Beverley A. Orser wrote the manuscript.

### Conflict of interest statement

The authors declare that the research was conducted in the absence of any commercial or financial relationships that could be construed as a potential conflict of interest.
